# Comparison of Reef Fish Survey Data Gathered by Open and Closed Circuit SCUBA Divers Reveals Differences in Areas With Higher Fishing Pressure

**DOI:** 10.1371/journal.pone.0167724

**Published:** 2016-12-09

**Authors:** Andrew E. Gray, Ivor D. Williams, Kostantinos A. Stamoulis, Raymond C. Boland, Kevin C. Lino, Brian B. Hauk, Jason C. Leonard, John J. Rooney, Jacob M. Asher, Keolohilani H. Lopes, Randall K. Kosaki

**Affiliations:** 1 Coral Reef Ecosystem Program, Ecosystem Sciences Division, Pacific Islands Fisheries Science Center, National Oceanic and Atmospheric Administration, Honolulu, Hawaii, United States of America; 2 Joint Institute for Marine and Atmospheric Research, University of Hawaii at Manoa, Honolulu, Hawaii, United States of America; 3 Department of Environment and Agriculture, Curtin University, Perth, Australia; 4 Fisheries Ecology Research Lab, Department of Biology, University of Hawaii at Manoa, Honolulu, Hawaii, United States of America; 5 Ecosystem Sciences Division, Pacific Islands Fisheries Science Center, National Oceanic and Atmospheric Administration, Honolulu, Hawaii, United States of America; 6 Papahānaumokuākea Marine National Monument, National Oceanic and Atmospheric Administration, Honolulu, Hawaii, United States of America; 7 Tropical Conservation Biology and Environmental Science, University of Hawaii at Hilo, Hilo, Hawaii, United States of America; Australian National University, AUSTRALIA

## Abstract

Visual survey by divers using open-circuit (OC) SCUBA is the most widely used approach to survey coral reef fishes. Therefore, it is important to quantify sources of bias in OC surveys, such as the possibility that avoidance of OC divers by fishes can lead to undercounting in areas where targeted species have come to associate divers with a risk of being speared. One potential way to reduce diver avoidance is to utilize closed circuit rebreathers (CCRs), which do not produce the noise and bubbles that are a major source of disturbance associated with OC diving. For this study, we conducted 66 paired OC and CCR fish surveys in the Main Hawaiian Islands at locations with relatively high, moderate, and light fishing pressure. We found no significant differences in biomass estimates between OC and CCR surveys when data were pooled across all sites, however there were differences at the most heavily fished location, Oahu. There, biomass estimates from OC divers were significantly lower for several targeted fish groups, including surgeonfishes, targeted wrasses, and snappers, as well as for all targeted fishes combined, with mean OC biomass between 32 and 68% of mean CCR biomass. There were no clear differences between OC and CCR biomass estimates for these groups at sites with moderate or low fishing pressure, or at any location for other targeted fish groups, including groupers, parrotfishes, and goatfishes. Bias associated with avoidance of OC divers at heavily fished locations could be substantially reduced, or at least calibrated for, by utilization of CCR. In addition to being affected by fishing pressure, the extent to which avoidance of OC divers is problematic for visual surveys varies greatly among taxa, and is likely to be highly influenced by the survey methodology and dimensions used.

## Introduction

Conducting underwater visual surveys using open-circuit (OC) SCUBA is a widely accepted method used to assess the status and structure of reef fish populations [1–4]. Studies largely based on data gathered by divers using SCUBA have shown dramatic effects of fishing on coral reef fish stocks, including large declines in biomass, reductions in mean size, alterations of sex ratios, and these effects can be substantial even at relatively low levels of fishing pressure [5–12]. There is, however, a growing awareness of potential biases relating to methodology and differences among observers, which can be consequential when assessing reef fish populations [13–16]. An additional potential source of bias, and the one we focus on here, is that the presence of divers can alter coral reef fish behavior in ways that could lead to under- or over-counting, with responses varying among species and depending on other factors, including whether divers are perceived as a threat [3, 17–23].

A major component of the disturbance associated with OC SCUBA diving is from the loud and conspicuous stream of bubbles produced when divers exhale [24]. Studies have shown fishes to be both attracted to and repelled by the sound of divers’ bubbles from beyond visual range–up to ~200 meters away [25]—in temperate, freshwater, and offshore environments [26–29]. One way to greatly reduce that source of disturbance is to use closed circuit rebreather (CCR) systems, which ordinarily do not release bubbles into the water [25, 30]. Although use of rebreathers appears to offer large potential benefits for visual surveys of some temperate fishes [31], we are only aware of one previous study by Lindfield et al. [32] that has compared use of OC and CCR for coral reef fish surveys. For that study, divers used stereo-video systems to conduct paired OC and CCR belt transect surveys of coral reef fish assemblages at a range of locations around Guam. Compared to CCR, OC divers recorded lower density and richness of targeted species at heavily fished sites presumably due to increased avoidance of OC divers by fishes at sites where they had come to associate OC SCUBA with a risk of being speared. There was, however, relatively little difference at reserve sites (closed to fishing) or at more lightly-fished sites. The net effect was that OC surveys greatly magnified the differences in target fish densities between reserve sites and heavily fished-sites [32].

As visual surveys by divers using OC is by far the most commonly used approach to survey coral reef fishes, it is critical to determine whether the OC SCUBA-bias shown by Lindfield et al. [32]is evident at other locations and using other underwater survey methods. To that end, we conducted a series of paired OC and CCR fish surveys in the Main Hawaiian Islands using a stationary point count visual survey method. Specifically, we compared estimated biomass of a range of targeted and non-targeted coral reef fishes at sites separated into 3 categories based on presumed fishing intensity (relatively high, medium, and low) to determine whether there are systematic differences between OC and CCR counts for specific taxa and whether those vary at different levels of fishing pressure.

## Materials and Methods

### Ethics Statement

Partner agencies that contributed to field personnel and equipment for this study were Papahānaumokuākea Marine National Monument, University of Hawaii Fisheries Ecology Research Lab, NOAA Diving Program, State of Hawaii Division of Aquatic Resources, and University of Hawaii Diving Safety Program. Authorization for research was given by the National Environmental Policy Act (NEPA): Programmatic Environmental Assessment for Research Activities Conducted by the Coral Reef Ecosystem Division, PIFSC, 2010–2015.

### Study Area and Survey Program

We conducted 66 paired stationary point count surveys using both OC SCUBA and CCR in coral reef habitats around seven of the Main Hawaiian Islands (Hawaii, Maui, Lanai, Molokai, Oahu, Kauai, and Niihau) from June 18 to August 13, 2015 at depths of ~12–30 meters. The majority of surveys were conducted during a routine NOAA Coral Reef Ecosystem Program (CREP) survey cruise as part of a larger monitoring effort [33]. All survey sites were on hard-bottom habitat (flat pavement, rock and boulder, and aggregate coral reef). Locations were randomly preselected using a geographic information system and habitat and depth strata maps maintained by CREP.

**Fig 1:**
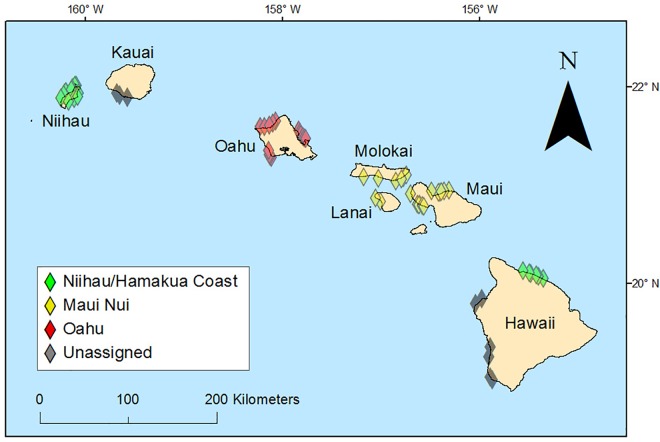
Map of survey locations. Diamonds mark locations of paired OC-CCR survey sites. The color of diamonds indicate presumed fishing pressure with red = high, yellow = medium and green = low.

In order to compare differences between OC and CCR fish surveys at different levels of human impact and presumed spearfishing pressure, sites were classified into ‘location groups’: ‘Oahu’ (n = 15); ‘Maui Nui’ (n = 20) and ‘Niihau-Hamakua’ (n = 22, Fig 1), representing presumed high, moderate, and low fishing pressure. In Hawaii, the great majority of fishing for coral reef fishes is non-commercial [34–36], typically for subsistence, recreation and/or sharing with wider social networks [35]. Expanded estimates of non-commercial fishery catch and effort expansions are generated by the Marine Recreational Information Program (MRIP), but those are only available at statewide level (i.e. for all of Hawaii), and thus it was not possible for us to quantify fishing effort or catch at the level of our study sites or locations. Therefore, presumed differences in fishing pressure among location groups were based on accessibility of survey sites to shore-based or boat-based fishers and on local population density using 2010 census data, converted to density per unit of reef area following methods described in Williams et al. [5]. Williams et al. [37] have previously shown large differences in target fish populations among those locations, and along a gradient of human population density in Hawaii, and attributed those to differences in fishing pressure. Specifically, the presumed low fishing pressure group included sites along the Hamakua Coast of Hawaii Island where nearshore reef areas are largely inaccessible due to high shoreline cliffs, limited road access, and long distances from nearest boat ramps or harbors [37]. These sites were pooled with sites around Niihau Island, which has a total population of 170 and the lowest human population density per reef area (2 people km^-2^ of forereef) of the inhabited islands in the Main Hawaiian Island group [5]. Sites around the island of Oahu were assumed to have relatively high fishing pressure, as Oahu is the most densely populated island in the Main Hawaiian Islands (3,795 people km^-2^ of forereef) and the majority of its reef area is highly accessible from shore and/or boat launches. Sites around Maui Nui were assumed to have fishing pressure somewhere between those extremes. Humans per reef area at those islands ranged between 58 and 1,299 people km^-2^ of reef. Nine sites (three off south Kauai and six off west Hawaii) did not naturally fit into any of those location groups and thus were only used in analysis only when all sites were pooled. It is important to recognize that the different location groups also had different dominant habitat types; Oahu sites were primarily located on flat areas of reef with patchy coral, Maui Nui sites typically had higher coral cover and more physical structure associated with that coral growth, and Niihau-Hamakua sites were primarily rock and boulder habitats with scattered coral.

Spearfishing is widely practiced in Hawaii, and is typically one of the main gear types used to target reef fishes. For example, small-scale CREEL surveys and fisher interviews in Hawaii have shown spearfishing to constitute between 8 and 41% of nearshore fishing effort at their study locations [34, 35, 38, 39]. SCUBA spearfishing is legal throughout the state of Hawaii, with the exception of 6 sites on the west side of Hawaii Island, where SCUBA spearfishing was banned in 2013. Across the state, spearfishing is mostly conducted by free divers or by divers using OC SCUBA gear. Within the depths covered by this study (12–30 m), we believe it is likely that the majority of spearfishing is by divers using OC SCUBA and we are not aware of any substantial degree of spearfishing using CCR.

### Dive Equipment

Open-circuit SCUBA surveys were conducted using conventional SCUBA equipment, which creates an intermittent flow of bubbles between the diver and the surface. CCR surveys were conducted using fully closed circuit rebreathers (Inspiration Vision: Ambient Pressure Diving Ltd. and Megalodon APECS 2.7: Innerspace Systems Corp.) that continuously recycle exhaled air through a breathing loop and only release bubbles on ascent. Electronic monitoring and controls add additional oxygen to the breathing loop to compensate for diver metabolic usage, while a chemical scrubber removes carbon dioxide (see review of CCR use for scientific diving by Seiber and Pyle [40]).

### Survey Methods

Standardized stationary point count (SPC) fish surveys were conducted at all locations. Details of the SPC method are available in Ayotte et al. [41]. In brief, divers work in pairs to record the number, size, and species of all fishes within adjacent, visually-estimated 15m-diameter cylinders along a 30 meter gray cotton transect line deployed at the start of the first survey dive. Divers spend the first five minutes of each survey listing fish species present within or passing though their cylinders. They then systematically work though the species list, recording the number, size, and species of all fishes present in the cylinder. Counting and sizing of species is done as a series of rapid visual sweeps, with each sweep focused on a different group of fishes (e.g. roving piscivores in one sweep, benthic-associated goatfish in another sweep, benthic butterflyfish in another sweep, and so on through all groups recorded). Divers record encounters with any additional species (i.e. those not recorded during the initial 5-minute period) that pass through their cylinder at any point during the survey when the species is first observed within their survey cylinder. Thus, total counts for a site are conducted as a series of focused counts on specific groups and include abundance and size information on all species present in or passing through the cylinder in the course of the survey (typically 25–30 minutes in length). For each species only one count is made, recorded as a snapshot of all fish of that species inside the survey cylinder. In cases where a species was present during the first 5 minutes, but not subsequently observed, survey divers record the number and sizes of fish of that species when they were first observed. Divers attempt to remain near the center of their cylinders (15m apart) for as much of the dive as possible, only moving to search through the cylinder to count small and cryptic species towards the end of the surveys. All sites were surveyed using both OC and CCR dive modes. To ensure the exact same area was surveyed, the first dive pair left the transect line attached to the bottom marked by a surface float before the second survey team entered the water approximately 5–15 minutes after completion of the first survey. However, the second survey team would re-swim along the transect line before beginning their survey to simulate the disturbance caused by laying the transect line in the first dive. Each survey team conducted surveys at 2–3 sites per day and the order of OC and CCR surveys alternated for each survey site. Fifteen experienced and highly trained divers participated, with six as CCR divers and nine as OC divers (but all CCR divers also participated as OC divers). In order to reduce scope for observer bias from any individuals, dive teams were interchanged daily resulting in 14 unique CCR dive teams and 22 unique OC dive teams.

### Fish Groupings

For analysis, fishes were categorized into groups based on behavioral characteristics, desirability to fishers, size, and vulnerability to spearfishing (S1 Table). Prior to analysis, ‘noisy species’ were identified as those that are generally rare but can form large schools (e.g. the blue-lined snapper *Lutjanus kasmira*), and including those typically encountered as transient schools high in the water column (e.g. planktivorous surgeonfishes, *Decapterus macarellus)*, or have very large biomass and are infrequently encountered (e.g. sharks, manta rays). Noisy species were excluded before estimation of biomass per fish groups. After removing noisy species, all species were classified as either fished or unfished. Fished species were those known to be commonly targeted in Hawaii or frequently recorded in catch data gathered by MRIP (http://www.st.nmfs.noaa.gov/recreational-fisheries/index), and include parrotfish, surgeonfish, goatfish (Mullidae), jacks (Carangidae), snappers (Lutjanidae), the one species of grouper encountered on shallow reefs (*Cephalopholis argus*), soldierfish and squirrelfish (Holocentridae), as well as a few other species (S1 Table). Fishes observed by divers were only classified as ‘fished’ if they were larger than a minimum target size, 20 cm total length (TL), except for two species of surgeonfishes (*Ctenochaetus strigosus* and *Acanthurus triostegus*) that are frequently targeted at relatively small size, and for which we set a minimum size of 15 cm TL (S1 Table). We further divided ‘unfished’ fishes into ‘unfished midwater’ and ‘unfished benthic,’ as bubbles moving upwards through the water column could directly displace mid-water species’ and alter their behavior and distribution [42] while benthic species might not be so directly affected. We did not separate ‘fished’ taxa into mid-water and benthic because none of those in our study aggregate in mid-water, i.e. they are all benthic associated or roving species. We further separated the ‘fished’ and ‘unfished’ categories based on family and behavior, with focal target groups being surgeonfishes (Acanthuridae) and parrotfishes (Labridae sub-family Scarinae), and focal non-target groupings being benthic triggerfish (Balistidae), non-planktivorous butterflyfish (Chaetodontidae), and non-target wrasses (Labridae).

To compare sightings of individual species between CCR and OC SCUBA surveys, we also generated a list of high interest and high target species (Table 1). These consist of species of special interest based on rarity, value to the recreational diving industry, and particularly, on desirability to spearfishers and aquarium collectors. Additionally, size frequency histograms were created to compare size distributions of fishes in target groups observed on CCR and OC surveys (S1 and S2 Figs).

**Table 1:** Total counts for all observed high interest and high target species at each location group and all locations. All locations includes Oahu (n = 15), Maui Nui (n = 20), Niihau-Hamakua (n = 22) West Hawaii (n = 6) and South Kauai (n = 3).

Taxon		Oahu		Maui Nui		Niihau-Hamakua		All Locations	
	* *	OC	CCR	OC	CCR	OC	CCR	OC	CCR
Acanthuridae	*Acanthurus blochii*	2	5	2	8	31	48	39	62
	*Acanthurus dussumieri*	-	5	8	9	44	42	57	65
	*Acanthurus nigroris*	4	3	10	9	17	10	31	23
	*Acanthurus olivaceus*	40	47	46	62	100	81	202	203
	*Acanthurus triostegus*	-	1	67	36	45	24	112	61
	*Ctenochaetus hawaiiensis*	-	-	2	3	-	4	33	28
	*Ctenochaetus strigosus*	48	29	541	546	74	71	895	928
	*Naso hexacanthus*	49	129	124	198	72	81	248	433
	*Naso lituratus*	39	38	30	42	36	32	141	161
	*Naso unicornis*	15	48	5	12	34	35	56	96
	*Zebrasoma flavescens*	11	9	136	215	25	25	389	455
Carangidae	*Carangoides orthogrammus*	-	2	4	2	2	2	6	6
	*Caranx ignobilis*	1	1	1	35	2	1	4	39
	*Caranx melampygus*	5	6	3	6	34	38	47	52
	*Decapterus macarellus*	42	42	23	37	153	507	218	1489
Elasmobranchii	*Aetobatus narinari*	-	3	1	1	-	-	2	4
	*Carcharhinus amblyrhynchos*	-	-	-	-	2	-	2	-
	*Manta spp*.	-	2	2	2	2	1	4	6
	*Triaenodon obesus*	1	-	-	-	1	4	2	4
Labridae	*Bodianus albotaeniatus*	22	21	38	44	70	57	141	131
Lethrinidae	*Monotaxis grandoculis*	2	2	38	27	44	41	87	78
Lutjanidae	*Aphareus furca*	2	1	4	2	20	18	32	33
	*Aprion virescens*	2	9	8	6	22	25	33	42
	*Lutjanus kasmira*	74	25	3	-	52	67	130	92
Mullidae	*Parupeneus cyclostomus*	19	11	6	10	31	18	58	45
	*Parupeneus insularis*	1	2	-	8	30	28	32	40
	*Parupeneus multifasciatus*	133	149	46	41	82	54	288	284
	*Parupeneus porphyreus*	-	-	-	-	5	6	5	6
Scaridae	*Calotomus carolinus*	6	9	5	3	13	13	25	32
	*Calotomus zonarchus*	4	3	1	2	6	3	13	8
	*Chlorurus perspicillatus*	-	-	4	1	4	2	8	3
	*Chlorurus spilurus*	6	18	188	212	8	2	225	251
	*Scarus psittacus*	11	27	49	34	45	3	115	71
	*Scarus rubroviolaceus*	13	4	23	47	36	35	76	88
Serranidae	*Cephalopholis argus*	3	3	20	27	26	22	56	63
	All species	555	654	1438	1687	1168	1400	3812	5382

### Data Analysis

Biomass was calculated by converting fish length to weight based on length-weight relationships available from Kulbicki 2004, Kulbicki 2005, or Froese 2015 [43–45]. Fish biomass per OC or CCR survey was generated by summing estimated fish biomass from the two divers and dividing by the combined area of their 2 cylinders (~353.4 m^2^). In order to compare between OC and CCR surveys, we calculated the differences in fish biomass between CCR and OC data at each site and for each response variable (i.e. each fish grouping). We used the boot and boot.ci routines from the boot package in R (1.3–17), with 10,000 iterations to generate the adjusted bootstrap 95% confidence intervals (type =“bca”) of those biomass differences. As a form of normalization among response variables and for improved visualization of difference between dive modes, we converted the values of absolute difference (i.e. CCR-OC) into OC:CCR ratios. Results are reported as OC:CCR biomass ratio [BR] together with 95% confidence intervals [95%CI] of those ratios. BRs with confidence intervals that do not overlap 1 indicate significantly greater values on either OC (95% CI >1), or on CCR (95% CI <1) at alpha of 0.05.

We assessed biomass differences between CCR and OC counts at all survey sites together and separately for the 3 location groupings representing presumed different levels of fishing pressure (‘Oahu’, ‘Maui Nui’ and ‘Niihau-Hamakua’).

As noted above, the order of surveys (i.e. whether OC or CCR survey was conducted first) was alternated between sites. To test for systematic differences relating to survey order, we used the same bootstrapping approach to generate mean and 95% confidence intervals of biomass differences between the first and second counts at each site. Of the 9 response groups analyzed separately for the 3 location groups and for all sites pooled the only significant difference was that estimated biomass of target surgeonfishes was higher on the first counts at sites in Niihau-Hamakua and for all sites pooled (S3 Fig). Notably, at the two locations we assumed to be more heavily fished (Oahu and Maui Nui) there is no indication of an impact of count order.

## Results

**Fig 2:**
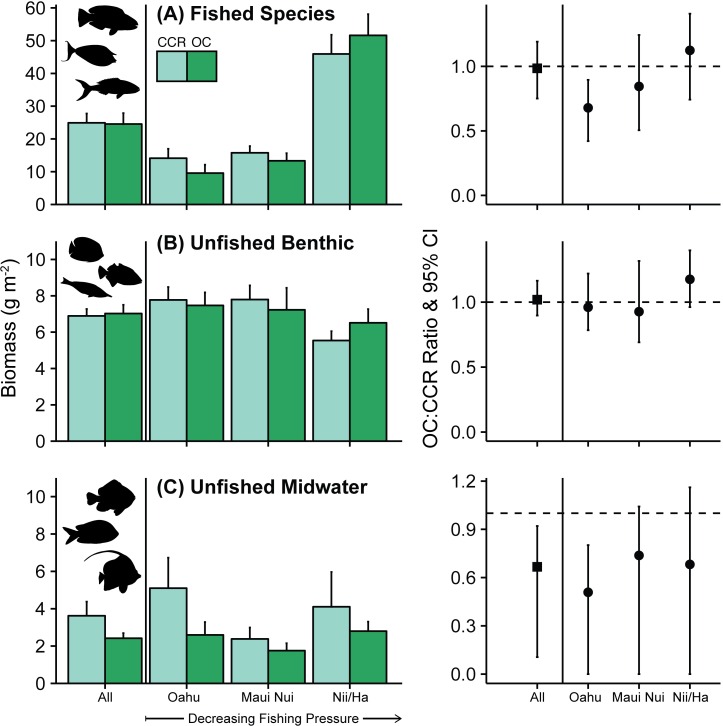
Fished and Unfished Biomass. Fished species ≥20 cm TL (except *A*. *triostegus* and *C*. *strigosus* ≥15 cm). Biomass boxplot with error bars indicating standard error for all sites and for Oahu, Maui Nui and Nii-Hama (Niihau and Hamakua coast). Location groups are ordered in direction of presumed declining spearfishing pressure from Oahu (highest) to Niihau-Hamakua (lowest). OC:CCR biomass ratio has 95% confidence interval for each location.

**Fig 3:**
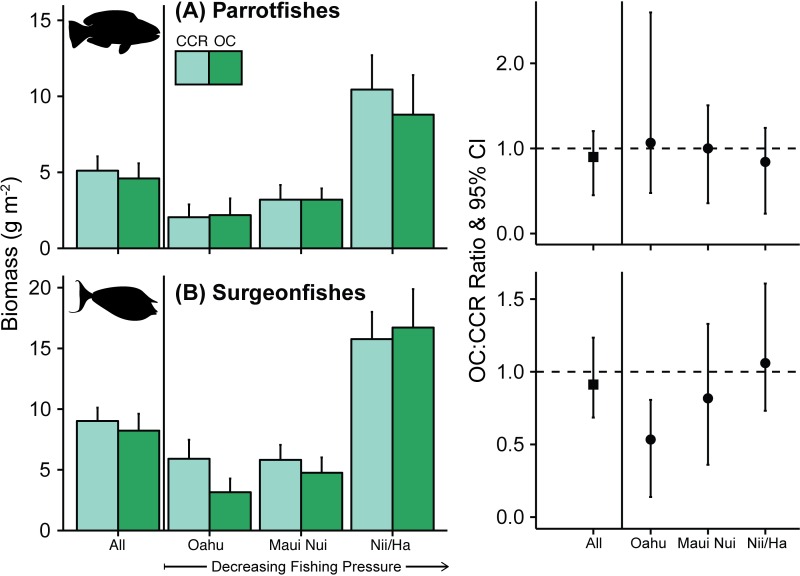
Biomass for target groups. Target fishes ≥20 cm TL (except *A*. *triostegus* and *C*. *strigosus* ≥15 cm). Biomass boxplot with error bars indicating standard error for all sites and for Oahu, Maui Nui and Nii-Hama (Niihau and Hamakua coast). Location groups are ordered in direction of presumed declining spearfishing pressure from Oahu (highest) to Niihau-Hamakua (lowest). OC:CCR biomass ratio has 95% confidence interval for each location.

**Fig 4:**
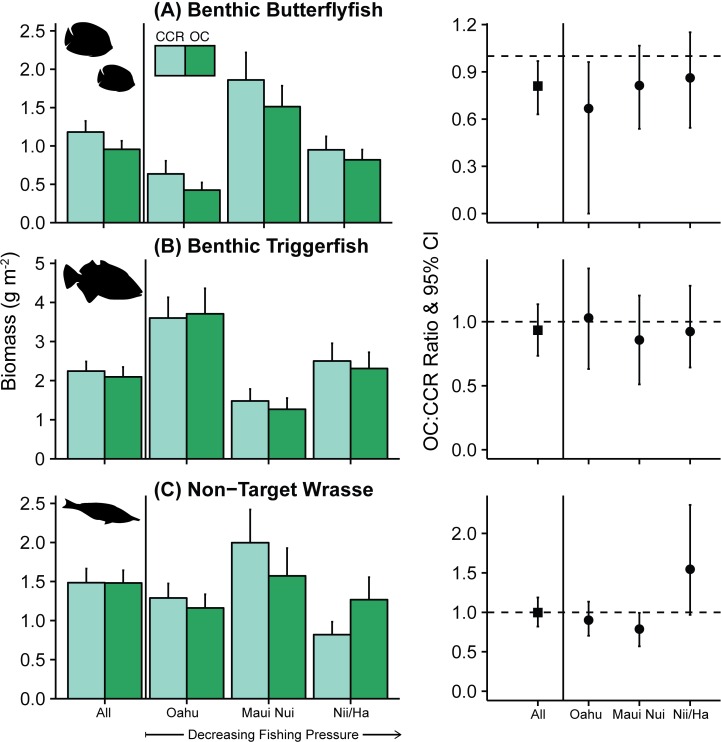
Biomass for non-target groups. Biomass boxplot with error bars indicating standard error for all sites and for Oahu, Maui Nui and Nii-Hama (Niihau and Hamakua coast). Location groups are ordered in direction of presumed declining spearfishing pressure from Oahu (highest) to Niihau-Hamakua (lowest). OC:CCR biomass ratio has 95% confidence interval for each location.

Across all sites pooled, differences between OC and CCR reef fish biomass estimates were mostly small (with OC:CCR biomass ratio [BR] being between 0.90 and 1.15), and non-significant; 95% confidence intervals overlapped 1.0 for all analyzed groups other than goatfishes, unfished-midwater fishes, and benthic butterflyfishes (Figs 2–4, S4 Fig, Tables 2 and 3). Biomass of benthic butterflyfishes and unfished mid-water fishes were lower on OC than CCR (BR 0.81, 95%CI: 0.63–0.97 and 0.67, 95%CI: 0.11–0.92 respectively, Table 3, Fig 4). Goatfish biomass tended to be higher on OC, although not substantially so, probably due to high variability in goatfish counts (BR 1.51, 95%CI: 0.91–2.38, Table 2, S4 Fig).

**Table 2:** Summary table for biomass (g/m^2^) of fished taxa. Fished taxa ≥20 cm TL (except *A*. *triostegus* and *C*. *strigosus* ≥15 cm). N = number of sites with the group observed, OC:CCR Ratio = ratio of mean OC value to mean CCR value with 95% confidence interval (low 95% CI, high 95% CI). Significant differences between CCR and OC (i.e. 95% CI of OC:CCR not overlapping 1) are marked with an asterisk (*) and shown in bold. Number of sites at each location was Oahu: n = 15, Maui Nui: n = 20, Niihau-Hamakua: n = 22 and all sites: n = 66. Location groups are listed in order of presumed declining spearfishing pressure from Oahu (highest) to Niihau-Hamakua (lowest).

FISHED TAXA	Location	OC			CCR			OC:CCR	
Group	Group	N	Mean	SE	N	Mean	SE	Ratio (95% CI)	
All Fished Taxa	ALL	62	24.54	3.33	64	24.91	2.83	0.99	(0.75, 1.19)
	Oahu	12	9.58	2.55	14	14.11	2.89	**0.68**	(0.42, 0.90) *
	Maui Nui	19	13.31	2.34	20	15.76	2.04	0.84	(0.51, 1.24)
	Nii-Hama	22	51.62	6.45	21	45.93	5.87	1.12	(0.74, 1.41)
Parrotfish	ALL	39	4.59	0.99	43	5.11	0.95	0.90	(0.45, 1.20)
	Oahu	5	2.18	1.10	9	2.04	0.84	1.07	(0.48, 2.60)
	Maui Nui	14	3.20	0.74	12	3.20	0.97	1.00	(0.36, 1.51)
	Nii-Hama	15	8.79	2.61	17	10.45	2.26	0.84	(0.23, 1.24)
Surgeonfish	ALL	54	8.23	1.39	58	9.02	1.10	0.91	(0.68, 1.23)
	Oahu	9	3.15	1.13	12	5.91	1.57	**0.53**	(0.14, 0.81) *
	Maui Nui	16	4.76	1.26	19	5.82	1.23	0.82	(0.36, 1.33)
	Nii-Hama	21	16.71	3.17	20	15.77	2.23	1.06	(0.73, 1.61)
Goatfishes	ALL	30	1.17	0.30	29	0.77	0.16	1.51	(0.91, 2.38)
	Oahu	3	0.77	0.55	4	0.58	0.29	1.33	(0.22, 3.17)
	Maui Nui	5	0.82	0.68	7	0.46	0.21	1.79	(0.31, 6.43)
	Nii-Hama	17	2.06	0.53	14	1.21	0.31	1.70	(0.88, 2.69)
Target Wrasse	ALL	48	1.81	0.26	47	2.02	0.31	0.89	(0.59, 1.20)
	Oahu	9	0.71	0.22	9	1.65	0.55	**0.43**	(0, 0.97) *
	Maui Nui	14	1.57	0.34	16	2.52	0.62	0.62	(0.14, 1.01)
	Nii-Hama	20	3.21	0.59	17	2.15	0.58	1.49	(0.98, 2.08)
Snapper	ALL	30	3.24	0.73	39	3.14	0.53	1.03	(0.63, 1.56)
	Oahu	2	0.66	0.57	7	2.06	0.74	**0.32**	(0, 0.92) *
	Maui Nui	4	0.92	0.55	5	0.97	0.50	0.95	(0, 2.62)
	Nii-Hama	21	8.26	1.67	20	6.59	1.13	1.25	(0.73, 1.90)
Grouper	ALL	30	1.74	0.49	34	1.52	0.25	1.15	(0.77, 1.91)
	Oahu	2	0.20	0.18	2	0.38	0.33	0.53	(0, 1.07)
	Maui Nui	10	1.49	0.50	14	1.66	0.36	0.89	(0.37, 1.80)
	Nii-Hama	13	3.41	1.33	13	2.20	0.56	1.55	(0.91, 3.03)

**Table 3:** Summary table for biomass (g/m^2^) of unfished taxa. N = number of sites with the group observed, OC:CCR Ratio = ratio of mean OC value to mean CCR value with 95% confidence interval (low 95% CI, high 95% CI). Significant differences between CCR and OC (i.e. 95% CI of OC:CCR not overlapping 1) are marked with an asterisk (*) and shown in bold. Number of sites at each location was Oahu: n = 15, Maui Nui: n = 20, Niihau-Hamakua: n = 22 and all sites: n = 66. Location groups are listed in order of presumed declining spearfishing pressure from Oahu (highest) to Niihau-Hamakua (lowest).

UNFISHED TAXA	Location	OC			CCR			OC:CCR	
Group	Group	N	Mean	SE	N	Mean	SE	Ratio (95% CI)	
Benthic	ALL	66	7.02	0.48	66	6.89	0.38	1.02	(0.90, 1.16)
	Oahu	15	7.47	0.72	15	7.77	0.71	0.96	(0.78, 1.22)
	Maui Nui	20	7.23	1.21	20	7.80	0.77	0.93	(0.69, 1.32)
	Nii-Hama	22	6.51	0.76	22	5.54	0.52	1.18	(0.96, 1.40)
Midwater	ALL	64	2.42	0.28	65	3.62	0.75	**0.67**	(0.11, 0.92) *
	Oahu	15	2.59	0.69	14	5.10	1.63	**0.51**	(0, 0.80) *
	Maui Nui	19	1.76	0.40	20	2.38	0.61	0.74	(0, 1.04)
	Nii-Hama	21	2.80	0.51	22	4.10	1.86	0.68	(0, 1.16)
Benthic Butterfly	ALL	57	0.96	0.11	60	1.18	0.15	**0.81**	(0.63, 0.97) *
	Oahu	13	0.42	0.10	13	0.64	0.17	**0.67**	(0, 0.96) *
	Maui Nui	18	1.51	0.27	19	1.86	0.36	0.81	(0.54, 1.07)
	Nii-Hama	18	0.82	0.13	20	0.95	0.17	0.86	(0.54, 1.15)
Triggerfish	ALL	65	2.10	0.25	65	2.24	0.25	0.93	(0.73, 1.14)
	Oahu	15	3.71	0.65	15	3.60	0.53	1.03	(0.63, 1.42)
	Maui Nui	19	1.27	0.29	20	1.48	0.31	0.86	(0.51, 1.20)
	Nii-Hama	22	2.31	0.42	22	2.50	0.45	0.92	(0.64, 1.28)
Non-target Wrasse	ALL	66	1.48	0.16	66	1.49	0.18	1.00	(0.82, 1.19)
	Oahu	15	1.16	0.17	15	1.29	0.19	0.90	(0.70, 1.13)
	Maui Nui	20	1.57	0.35	20	2.00	0.42	**0.79**	(0.57, 0.99) *
	Nii-Hama	22	1.27	0.29	22	0.82	0.16	1.55	(0.97, 2.36)

### OC-CCR differences for fishery target and non-target groups

Analyzing OC-CCR differences by location group, i.e. separately for Oahu, Maui Nui, and Niihau-Hamakua (which we assume are relatively high, moderate, and low fishing pressure locations, respectively), revealed clear differences for several fish groupings at Oahu sites. This includes the Oahu estimates of total fished species biomass by OC divers, which were around one third lower than those by CCR divers (BR = 0.68, 95%CI: 0.42–0.90, Table 2 and Fig 2). In contrast, there were no significant differences for those at Maui Nui (BR 0.84, 95%CI: 0.51–1.25) or Niihau-Hamakua (BR: 1.12, 95%CI: 0.74–1.41).

Total unfished benthic biomass did not significantly differ between OC and CCR at any location group (Fig 2). However, unfished midwater species biomass was significantly lower at Oahu using OC (BR: 0.51, 95%CI: 0–0.80), and tended to be lower, although non-significantly, at both Maui Nui and Niihau-Hamakua (Fig 2).

### OC-CCR differences by family grouping

Among primary target taxa, parrotfish biomass did not significantly differ between the two diving modes at any location group (Fig 3), whereas surgeonfish biomass on OC at Oahu was approximately half of what was recorded on CCR (BR: 0.53, 95%CI: 0.14–0.81). Surgeonfish biomass did not differ significantly between OC and CCR at either Maui Nui or Niihau-Hamakua, where biomass ratios were 0.82 and 1.06 respectively (Table 2, Fig 2).

Other targeted taxa–goatfishes, target wrasse, snapper, and grouper–were infrequently observed at Oahu on either OC or CCR (Table 2). OC biomass was significantly lower than CCR biomass for two of those groups: target wrasse (BR: 0.43, 95%CI: 0–0.97, Table 2, S4 Fig) and snapper (BR: 0.32, 95%CI: 0–0.92, Table 2, S4 Fig). Goatfish biomass tended to be higher on OC at all location groups, with OC:CCR BR being between 1.33 and 1.79; however, none of those differences was significant (Table 2, S4 Fig). In fact, there were no significant differences between OC and CCR biomass for any target group at either Maui Nui or Niihau-Hamakua.

There were significant differences between CCR and OC for two other non-target family groups. For non-target wrasse, OC biomass was 21% lower than CCR biomass at Maui Nui (BR: 0.79, 95%CI: 0.57–0.99), but nearly significantly higher at Niihau-Hamakua (BR: 1.55, 95%CI: 0.97–2.36), showing no clear pattern relating to fishing pressure. Benthic butterflyfish biomass was 33% lower on OC at Oahu (BR: 0.67, 95%CI: 0–0.96).

### High interest and high target species

For most high interest and high target species, there was relatively little difference in total numbers observed by the two methods across all sites combined (Table 1). Exceptions were the surgeonfish *Naso unicornis* (96 recorded using CCR, 56 using OC), *Caranx ignobilis* (39 using CCR, 4 using OC, although 35 of those were at one site), two mid-water planktivores, *Naso hexacanthus* (433 recorded using CCR, 248 using OC), and *Decapterus macerellus* (1489 recorded using CCR, 218 using OC). For both *N*. *unicornis* and *N*. *hexacanthus*, CCR-OC differences were relatively clear at Oahu, but there was little difference in the number counted at Niihau-Hamakua sites (Table 1).

## Discussion

We found few differences between OC and CCR surveys for most fish groups when all sites were pooled together. However, analyzing the data separately for different levels of perceived spearfishing pressure revealed clear differences between OC and CCR survey results for several fish groupings. These differences were primarily found at sites around Oahu, the most heavily populated, and we presume most fished location, with few such differences at the other two location groups. In that respect, our results are consistent with those of Lindfield et al. [32] who found clear differences between CCR and OC counts at heavily-fished sites around Guam but not more lightly-fished sites. However, as we note above, there were also habitat differences among our study locations–Oahu sites generally being in low relief habitat with patchy coral, whereas Niihau and Hamakua sites were primarily in rock and boulder areas. Thus, we cannot rule out the possibility that some portion of the differences we found among locations reflected different wariness of fishes to divers in different reef type and/or different effects of diver avoidance on detectability in those different habitats.

Irrespective of acquired behavioral differences in relation to local spearfishing pressure, it has long been recognized that some taxa have generally predictable responses to divers, with some being relatively shy and others curious or indifferent [4, 19]. Thus, it is unsurprising that we saw clear differences in responses to OC divers of different target groups. Specifically, around Oahu (the most heavily fished location) we found evidence of OC-avoidance (lower estimated biomass) for surgeonfishes, snappers, and target wrasses but no indication of a difference for parrotfishes or targeted goatfishes. Furthermore, mean biomass of targeted goatfishes was actually higher when using OC than for CCR at all study locations, even if those effects were not significant at any location group. That not all effects are related to vulnerability to spearfishing is further illustrated by responses to OC divers among non-target groups, including unfished midwater species, which appeared to avoid OC divers at all locations, and benthic butterflyfishes that tended to avoid OC divers at Oahu. Butterflyfishes’ apparent wariness of OC divers around Oahu may reflect the fact that distinctions between target and non-target fishes are somewhat over-simplistic, particularly around Oahu, where depletion of target species may have led to fishers becoming less selective [46, 47]. Clearly, there are also differences within families, for example, contrary to the overall pattern for surgeonfishes, we saw no evidence that several of those (including *Acanthurus nigroris*, *A*. *olivaceus*, *Naso lituratus*, and *Ctenochaetus strigosus*) were more likely to avoid OC divers; whereas there were large differences for *Naso unicornis* (Table 1). As well as being known to be shy of divers [19], *N*. *unicornis* are a prized spearfishing target in Hawaii; therefore, it was unsurprising they were among the species that responded most clearly to the disturbance associated with OC-diving.

It was also notable that biomass estimates tended to be higher using OC for several fish groups at our most lightly fished study sites (Niihau-Hamakua), although none of those effects were significant. Attraction of roving predatory fishes—sharks and jacks—to divers on remote reefs in the Hawaiian Archipelago is a phenomenon that is known to cause substantial overestimation of their densities in small-scale surveys in the Northwestern Hawaiian Islands [48]. Although lightly populated and/or relatively inaccessible, Niihau and the Hamakua coast are much less isolated than reefs in the Northwestern Hawaiian Islands but the degree of spearfishing there may be low enough that OC SCUBA divers are not generally perceived as a danger, and thus slightly higher counts there might reflect responses of curious or opportunistic species.

Given the potential for bias due to OC-avoidance, it is important to consider the mechanisms by which increased diver avoidance might affect survey counts and, particularly, whether careful choice of methods can reduce the impact of those behaviors on survey counts. Broadly, diver-avoidance behavior that could lead to biased counts when amplified at heavily fished locations includes fishes moving completely out of an area before divers can begin a survey (‘fleeing’), as well as much smaller-scale effects, such as ‘hiding’ (becoming more cryptic) and small-scale ‘displacement’ (i.e. altering position or direction of swimming to avoid close proximity to divers). The first of those behaviors–fleeing–is clearly problematic for any underwater visual survey, and also one that is inherently difficult to quantify by in-water divers [26, 28]. As the sound of divers’ bubbles can be perceived over much greater distances (100s of meters) [25] than fishes can realistically be seen by divers (few 10s of meters or less), it seems unlikely that OC SCUBA divers can do much to reduce this potential source of bias, other than perhaps to incorporate waiting periods before beginning surveys [13]. In contrast, the impacts of hiding and displacement during survey counts will likely be highly dependent on the methodology and dimensions, such as the extent to which the method used allows divers to perceive hidden and partially hidden fishes. An important aspect of this is the scale over which displacement effects typically occur. Results from studies of ‘flight initiation distance’ (FID), in which divers swim purposefully towards fishes and measure the distance at which a flight response is initiated, consistently show elevated FID at heavily fished sites. However, for most taxa, FID tends to be small: typically ~1–3 m at unfished areas, increasing by 1–2 m at heavily-fished sites [17, 18, 20–22]. Distance sampling studies conducted by Kulbicki and colleagues also indicates that relatively small-scale displacement is a common response to divers–specifically, fish densities tend to peak a few meters away from surveyors as a consequence of fishes avoiding the area immediately around divers. Those density peaks tend to be further from divers in heavily fished locations, although rarely more than ~5–7 m [3, 19, 49]. The relatively small distance at which most target fishes react to divers’ approach and small scales of displacement in those FID and distance sampling studies may reflect the limited range over which fishes are vulnerable to capture by spear fishers, estimated as a little over 3 m by Feary et al. [20]. Thus, survey methods which involve counting fishes within a few meters of divers may be more prone to large effects from displacement than methods where survey boundaries are relatively far from divers (> 5–7 m) since displaced fishes would tend to remain within the survey unit dimensions.

Other research shows that some components of diver-avoidance effects appear to be short lived. For example, adding even a 5-minute waiting period before beginning a survey, during which divers remain relatively inactive, has been found to lead to substantially higher counts [13]. Relatively rapid acclimation to the presence of divers that are not perceived as a threat could mean that survey methods that involve stationary or slow moving divers and a short waiting period before beginning counts will tend to minimize avoidance behaviors and their impacts on counts. Conversely, moving divers, capable of surveying quite far ahead of themselves, might be able to count fishes before avoidance behavior is initiated [20].

Collectively, specific methods and survey dimensions are likely to have a substantial effect on the degree to which avoidance of divers using OC SCUBA could bias counts. The recent study by Lindfield et al. [32] is an important one because it provides clear evidence that, at least in some circumstances, bias caused by fishes’ avoidance of divers can be substantial enough to lead to erroneous conclusions. However, a clear difference between our results and theirs’ is in the scale of the OC-avoidance effect. They reported estimated biomass of targeted fishes at fished sites to be 200–300% higher when using CCR, whereas in our study, mean biomass from CCR surveys at Oahu sites was only around 50% higher than from OC surveys. That different scale of results might relate to inherent differences in habitat, fishing pressure, or in taxonomic composition between studies and locations. However, it is also possible that the difference is related to survey methodology. Our surveys were conducted by stationary divers in 7.5 m radius cylinders whereas theirs was a moving stereo-video (stereo-DOV) belt transect method inside a moving window of 10 m ahead and 2.5 m either side of the diver. While there are clear benefits to the stereo-video approach, including extremely accurate and precisely sized fish measurements [50] it seems likely that video gathered by a moving diver pointing the stereo-DOV unit straight ahead gives a more limited view of a surveyed portion of reef than is available to an in-situ diver performing a count, so that behaviors such as hiding, or when mid-water fishes move a little further off the substrate [51] which could be readily perceived in a visual survey, would potentially move fishes out of the field of view of the stereo-DOV. There is certainly a need for further comparative OC-CCR studies using different methodologies and survey dimensions and it would be wrong to generalize about the typical scale of OC-avoidance effects.

In addition to reduced disturbance due to the lack of bubbles and noise, advantages of CCRs over OC include extended no-decompression bottom times, shorter decompression times, and more efficient gas management [40, 52]. CCRs are particularly suitable for surveys in relatively deep water, where bottom time tends to be a limitation for OC divers, e.g. > ~30 m [53–58], but those advantages become less important in shallower water, and CCRs may be unrealistic for surveys shallower than ~5 meters. There are a number of major obstacles to the wider adoption of CCRs, including that they are more expensive to acquire than OC, require specialized training, and have considerably greater daily maintenance requirements and costs. Given those constraints, it may not be feasible for CCRs to be routinely utilized for fish surveys, even in heavily spearfished locations where they would be most beneficial. Over time, it may be possible to develop calibration coefficients suitable for particular methods and locations. In the meantime, those who gather and use visual survey data should recognize this possible source of bias that would tend to exaggerate the differences between heavily fished and more remote locations. In our study, surveys using OC would lead us to conclude that target species biomass around Oahu was 19% of that at the lightly-fished sites in Niihau-Hamakua, whereas surveys using CCR found Oahu biomass to be 31% of the value at Niihau-Hamakua.

In summary, we found clear evidence that OC-avoidance reduced biomass estimates of several groups of targeted fishes from visual surveys at sites around Oahu, but little evidence of systematic differences between OC and CCR survey results at our other two study location groups, Maui Nui and Niihau-Hamakua. Oahu and Guam, where the recent study by Lindfield et al. [32] also showed evidence of OC-avoidance, are both relatively heavily populated, have strong fishing cultures, and permit SCUBA-spearing [59, 60]. Together, our results indicate that there is certainly scope for OC-avoidance to lead to underestimation of target fish biomass, particularly at such heavily-fished locations. Therefore, where the operational and logistical challenges can be overcome, it may be desirable for survey programs to more routinely use, or at least compare, results from CCR surveys at heavily OC spearfished locations. Considerably more work is needed to properly understand the potential for a range of possible biases associated with in-water surveys, including OC-avoidance, as well as associated effects such as the presence and visible silhouette of a diver, and frequently the noise and disturbance caused by small boats used as dive platforms. For OC-avoidance, key topics include how that potential bias is mediated by survey methods and dimensions, the habitat in which surveys are conducted, the taxa being counted, as well as the type and degree of local fishing.

## Supporting Information

S1 Fig Histograms of total length for all parrotfish and target surgeonfish observed. Vertical lines indicate median values.
(TIF)

S2 Fig Histograms of total length for all target wrasses, target snappers, target goatfishes and groupers observed. Vertical lines indicate median values.
(TIF)

S3 Fig Survey order effect. Estimated biomass difference between the first and second surveys (OC then CCR or CCR then OC) as a proportion of CCR mean biomass with 95% confidence intervals.
(TIF)

S4 Fig Biomass for additional target groups. Target species ≥20 cm TL. Biomass boxplot with error bars indicating standard error for all sites and for Oahu, Maui Nui, and Nii-Hama (Niihau and Hamakua coast). Location groups are ordered in direction of presumed declining spearfishing pressure from Oahu (highest) to Niihau-Hamakua (lowest). OC:CCR biomass ratio has 95% confidence interval for each location. Encounter rates are low at Oahu for snapper, grouper and goatfish (see Table 2).
(TIF)

S1 File OC and CCR biomass data by site and method (XLSX)

S1 Table List of all species identified in surveys and corresponding status and group. Asterisks identify high interest species.
(PDF)
