# Supplementary figures and images for: Comparison of Reef Fish Survey Data Gathered by Open and Closed Circuit SCUBA Divers Reveals Differences in Areas With Higher Fishing Pressure
Source: PLoS One. 2016 Dec 9;11(12):e0167724. doi: 10.1371/journal.pone.0167724 (PMC5147984; doi:10.1371/journal.pone.0167724)

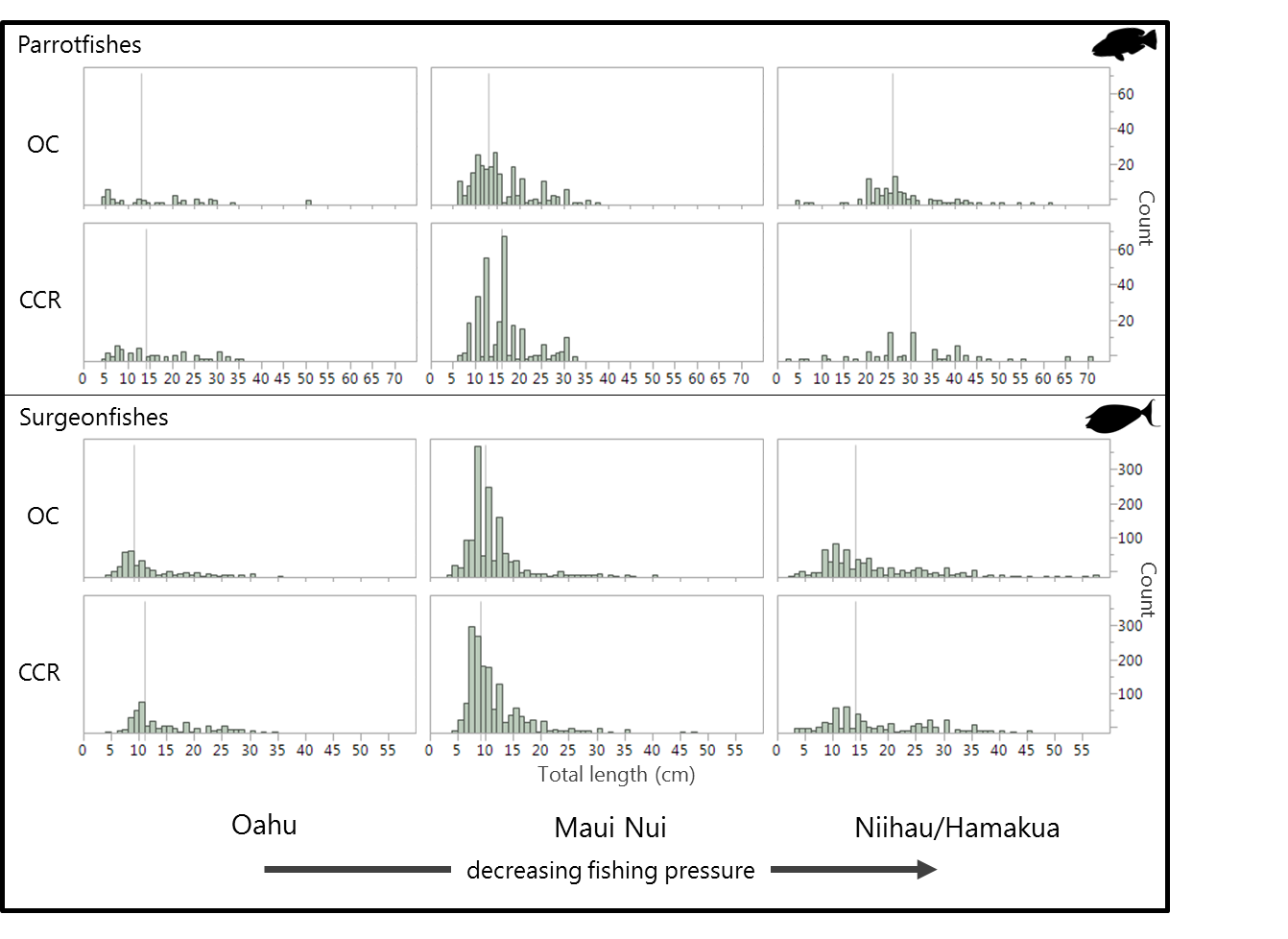

Supplement: S1 Fig — Vertical lines indicate median values. (TIF) [file pone.0167724.s001.tif]

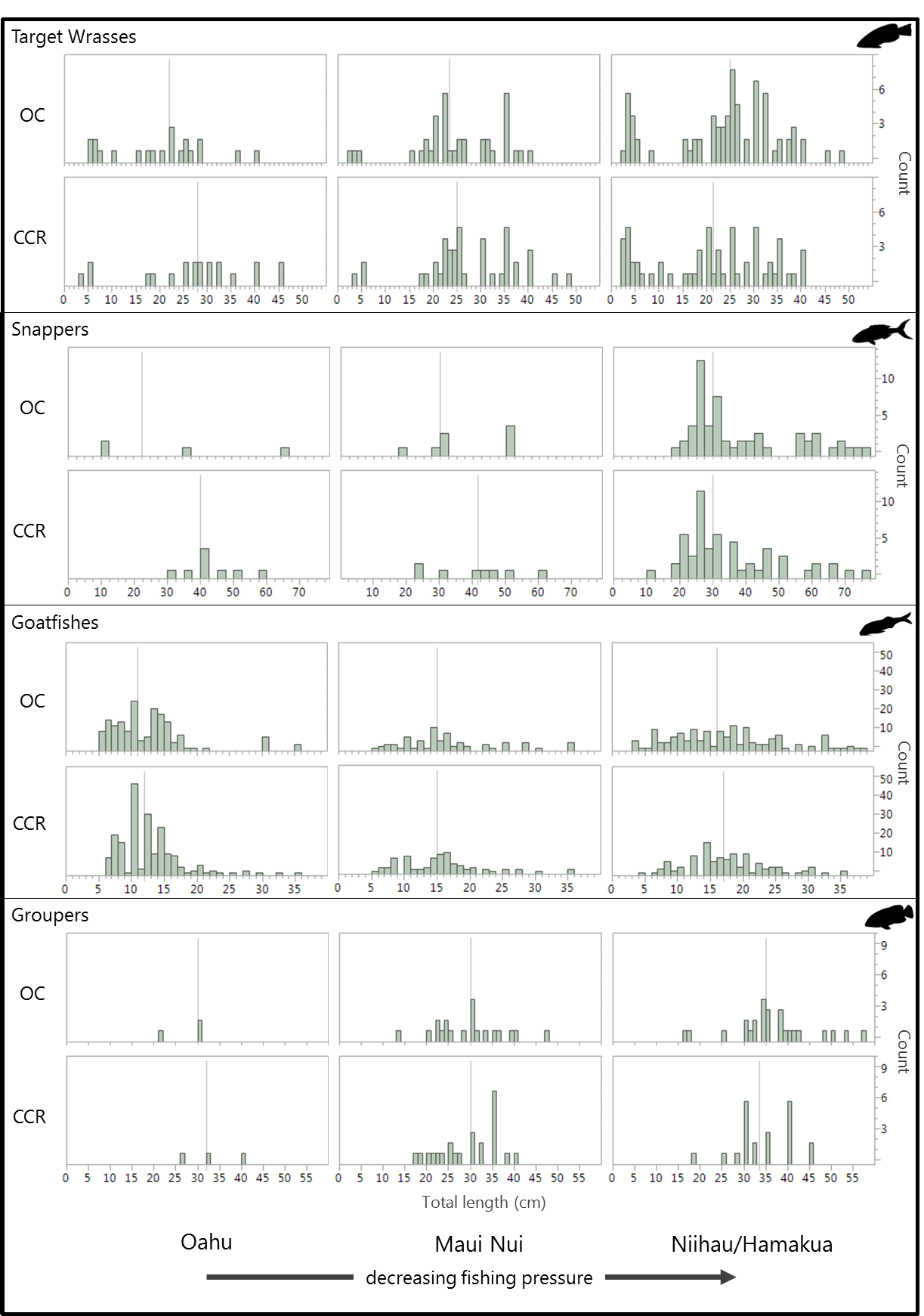

Supplement: S2 Fig — Vertical lines indicate median values. (TIF) [file pone.0167724.s002.tif]

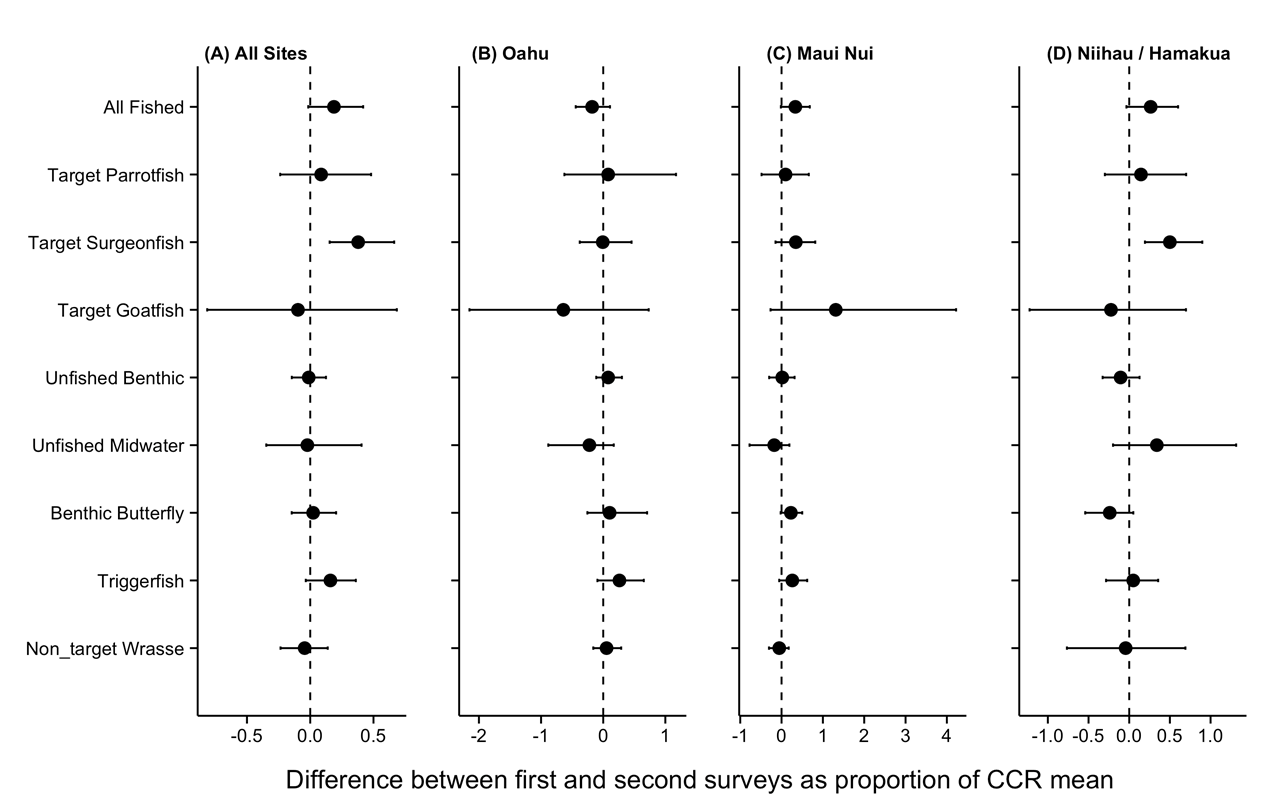

Supplement: S3 Fig — Estimated biomass difference between the first and second surveys (OC then CCR or CCR then OC) as a proportion of CCR mean biomass with 95% confidence intervals. (TIF) [file pone.0167724.s003.tif]

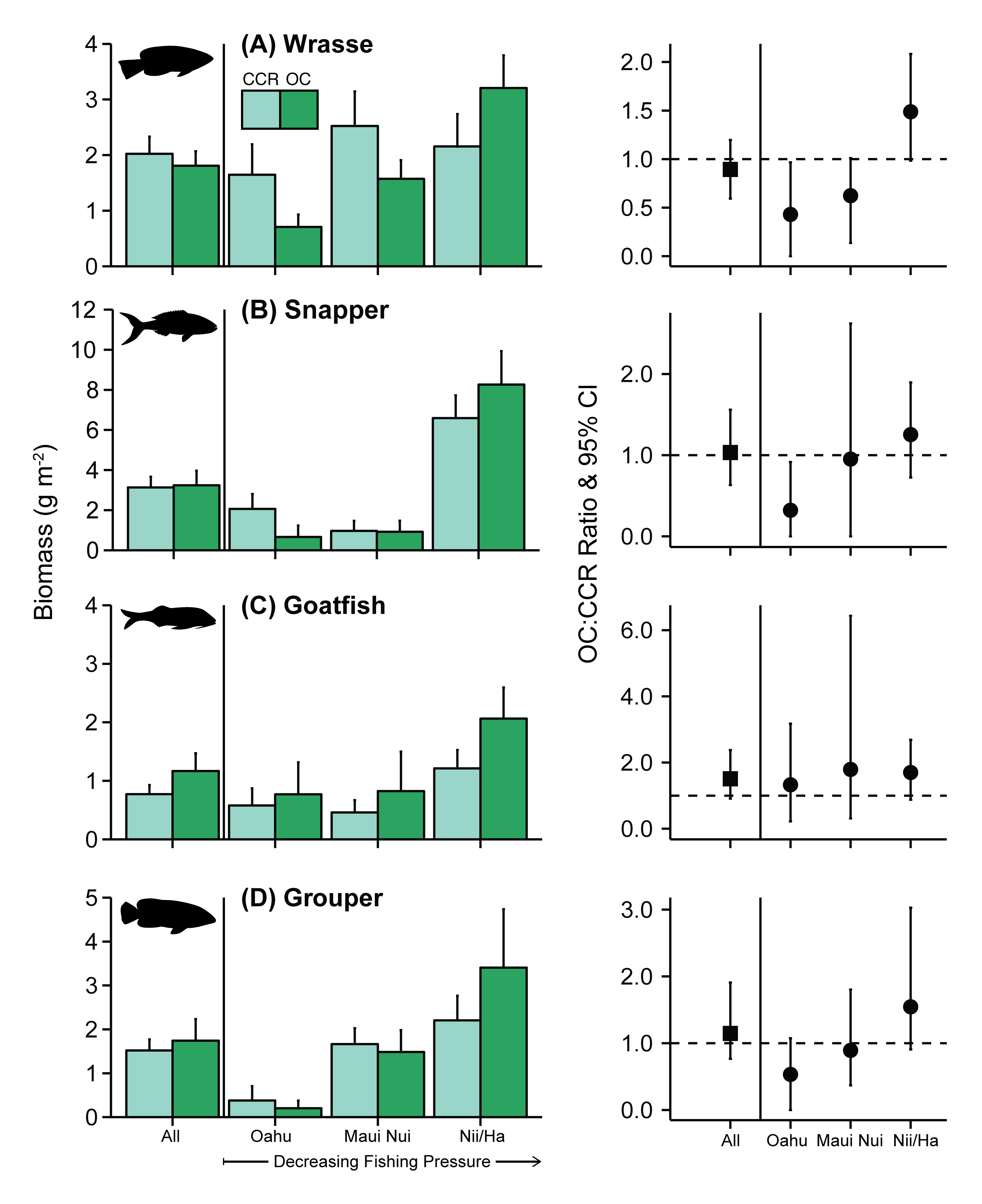

Supplement: S4 Fig — Target species ≥20 cm TL. Biomass boxplot with error bars indicating standard error for all sites and for Oahu, Maui Nui, and Nii-Hama (Niihau and Hamakua coast). Location groups are ordered in direction of presumed declining spearfishing pressure from Oahu (highest) to Niihau-Hamakua (lowest). OC:CCR biomass ratio has 95% confidence interval for each location. Encounter rates are low at Oahu for snapper, grouper and goatfish (see Table 2). (TIF) [file pone.0167724.s004.tif]
