# Supplementary material for: Comparison of Reef Fish Survey Data Gathered by Open and Closed Circuit SCUBA Divers Reveals Differences in Areas With Higher Fishing Pressure
Source: PLoS One. 2016 Dec 9;11(12):e0167724. doi: 10.1371/journal.pone.0167724 (PMC5147984; doi:10.1371/journal.pone.0167724)
Supplement: S1 Table — Asterisks identify high interest species. (PDF) [file pone.0167724.s006.pdf]

Family		Species	Status	Group	Minimum size (cm)
Acanthuridae		*Acanthuridae*	fished	surgeonfish	20
		Acanthurus achilles	fished	surgeonfish	20
	*	*Acanthurus blochii*	fished	surgeonfish	20
	*	*Acanthurus dussumieri*	fished	surgeonfish	20
		Acanthurus guttatus	fished	surgeonfish	20
		Acanthurus leucopareius	fished	surgeonfish	20
		Acanthurus nigricans	fished	surgeonfish	20
		Acanthurus nigrofuscus	fished	surgeonfish	20
	*	*Acanthurus nigroris*	fished	surgeonfish	20
	*	*Acanthurus olivaceus*	fished	surgeonfish	20
		Acanthurus sp	fished	surgeonfish	20
		Acanthurus thompsoni	noisy	-	-
	*	*Acanthurus triostegus*	fished	surgeonfish	15
		Acanthurus xanthopterus	fished	surgeonfish	20
	*	*Ctenochaetus hawaiiensis*	fished	surgeonfish	20
	*	*Ctenochaetus strigosus*	fished	surgeonfish	15
		Naso annulatus	noisy	-	-
		Naso brevirostris	noisy	-	-
	*	*Naso hexacanthus*	noisy	-	-
	*	*Naso lituratus*	fished	surgeonfish	20
	*	*Naso unicornis*	fished	surgeonfish	20
		Zebrasoma flavescens	fished	surgeonfish	20
		Zebrasoma veliferum	fished	surgeonfish	20
Apogonidae		*Apogonidae*	unfished benthic	-	-
Aulostomidae		*Aulostomus chinensis*	unfished benthic	-	-
Balistidae		*Balistes polylepis*	unfished benthic	triggerfish	-
		Balistidae	unfished benthic	triggerfish	-
		Melichthys niger	noisy	-	-
		Melichthys vidua	unfished midwater	-	-
		Rhinecanthus rectangulus	unfished benthic	triggerfish	-
		Sufflamen bursa	unfished benthic	triggerfish	-
		Sufflamen fraenatum	unfished benthic	triggerfish	-
		Xanthichthys auromarginatus	unfished midwater	-	-
		Xanthichthys caeruleolineatus	unfished midwater	-	-
Blenniidae		*Blenniidae*	unfished benthic	-	-
Caracanthidae		*Caracanthus typicus*	unfished benthic	-	-
Carangidae		*Carangoides ferdau*	fished	-	-
	*	*Carangoides orthogrammus*	fished	-	-
	*	*Caranx ignobilis*	noisy	-	-
	*	*Caranx melampygus*	fished	-	-
	*	*Decapterus macarellus*	noisy	-	-
		Scomberoides lysan	fished	-	-

		Seriola dumerili	fished	-	-
Carcharhinidae	*	*Carcharhinus amblyrhynchos*	noisy	-	-
	*	*Triaenodon obesus*	noisy	-	-
Chaetodontidae		*Chaetodon auriga*	unfished benthic	butterflyfish	-
		Chaetodon ephippium	unfished benthic	butterflyfish	-
		Chaetodon fremblii	unfished benthic	butterflyfish	-
		Chaetodon kleinii	unfished midwater	-	-
		Chaetodon lineolatus	unfished benthic	butterflyfish	-
		Chaetodon lunula	unfished benthic	butterflyfish	-
		Chaetodon lunulatus	unfished benthic	butterflyfish	-
		Chaetodon miliaris	unfished midwater	-	-
		Chaetodon multicinctus	unfished benthic	butterflyfish	-
		Chaetodon ornatissimus	unfished benthic	butterflyfish	-
		Chaetodon quadrimaculatus	unfished benthic	butterflyfish	-
		Chaetodon reticulatus	unfished benthic	butterflyfish	-
		Chaetodon unimaculatus	unfished benthic	butterflyfish	-
		Forcipiger flavissimus	unfished benthic	butterflyfish	-
		Forcipiger longirostris	unfished benthic	butterflyfish	-
		Hemitaurichthys polylepis	unfished midwater	-	-
		Heniochus diphreutes	unfished midwater	-	-
Cirrhitidae		*Amblycirrhitus bimacula*	unfished benthic	-	-
		Cirrhitops fasciatus	unfished benthic	-	-
		Cirrhitus pinnulatus	unfished benthic	-	-
		Paracirrhites arcatus	unfished benthic	-	-
		Paracirrhites forsteri	unfished benthic	-	-
Dasyatidae		*Dasyatis lata*	noisy	-	-
Diodontidae		*Diodon hystrix*	unfished benthic	-	-
Fistulariidae		*Fistularia commersonii*	unfished benthic	-	-
Gobiidae		*Gobiidae*	unfished benthic	-	-
Holocentridae		*Myripristinae*	fished	-	-
		Myripristis amaena	fished	-	-
		Myripristis berndti	fished	-	-
		Myripristis kuntee	fished	-	-
		Neoniphon sammara	fished	-	-
		Neoniphon sp	fished	-	-
		Sargocentron diadema	fished	-	-
		Sargocentron sp	fished	-	-
		Sargocentron tiere	fished	-	-
		Sargocentron xantherythrum	fished	-	-
Kyphosidae		*Kyphosus sp*	noisy	-	-
Labridae		*Anampses chrysocephalus*	unfished benthic	non-target wrasse	-
		Anampses cuvier	unfished benthic	non-target wrasse	-
		Anampses sp	unfished benthic	non-target wrasse	-
	*	*Bodianus bilunulatus*	fished	target wrasse	20
		Cheilio inermis	unfished benthic	non-target wrasse	-

		Cirrhilabrus jordani	unfished benthic	non-target wrasse	-
		Coris ballieui	unfished benthic	non-target wrasse	-
		Coris flavovittata	fished	target wrasse	20
		Coris gaimard	unfished benthic	non-target wrasse	-
		Coris venusta	unfished benthic	non-target wrasse	-
		Gomphosus varius	unfished benthic	non-target wrasse	-
		Halichoeres ornatissimus	unfished benthic	non-target wrasse	-
		Labridae	unfished benthic	non-target wrasse	-
		Labroides phthirophagus	unfished benthic	non-target wrasse	-
		Macropharyngodon geoffroy	unfished benthic	non-target wrasse	-
		Novaculichthys taeniourus	unfished benthic	non-target wrasse	-
		Oxycheilinus bimaculatus	unfished benthic	non-target wrasse	-
		Oxycheilinus unifasciatus	unfished benthic	non-target wrasse	-
		Pseudocheilinus evanidus	unfished benthic	non-target wrasse	-
		Pseudocheilinus octotaenia	unfished benthic	non-target wrasse	-
		Pseudocheilinus tetrataenia	unfished benthic	non-target wrasse	-
		Pseudojuloides cerasinus	unfished benthic	non-target wrasse	-
		Stethojulis balteata	unfished benthic	non-target wrasse	-
		Thalassoma ballieui	unfished benthic	non-target wrasse	-
		Thalassoma duperrey	unfished benthic	non-target wrasse	-
		Thalassoma sp	unfished benthic	non-target wrasse	-
Lethrinidae	*	*Monotaxis grandoculis*	fished	-	-
Lutjanidae	*	*Aphareus furca*	fished	snapper	20
	*	*Aprion virescens*	fished	snapper	20
	*	*Lutjanus fulvus*	fished	snapper	20
	*	*Lutjanus kasmira*	noisy	-	-
Malacanthidae		*Malacanthus brevirostris*	unfished benthic	-	-
Microdesmidae		*Gunnellichthys curiosus*	unfished benthic	-	-
Monacanthidae		*Aluterus scriptus*	unfished benthic	-	-
		Cantherhines dumerilii	unfished benthic	-	-
		Cantherhines pardalis	unfished benthic	-	-
		Cantherhines sandwichiensis	unfished benthic	-	-
		Cantherhines verecundus	unfished benthic	-	-
		Pervagor aspricaudus	unfished benthic	-	-
		Pervagor spilosoma	unfished benthic	-	-
Mullidae		*Mulloidichthys flavolineatus*	noisy	-	-
		Mulloidichthys pfluegeri	fished	goatfish	20
		Mulloidichthys vanicolensis	noisy	-	-
	*	*Parupeneus cyclostomus*	fished	goatfish	20
	*	*Parupeneus insularis*	fished	goatfish	20
	*	*Parupeneus multifasciatus*	fished	goatfish	20
		Parupeneus pleurostigma	fished	goatfish	20
	*	*Parupeneus porphyreus*	fished	goatfish	20
Muraenidae		*Gymnothorax eurostus*	unfished benthic	-	-
		Gymnothorax flavimarginatus	unfished benthic	-	-

		Gymnothorax javanicus	unfished benthic	-	-
		Gymnothorax melatremus	unfished benthic	-	-
		Gymnothorax meleagris	unfished benthic	-	-
		Gymnothorax rueppellii	unfished benthic	-	-
		Gymnothorax sp	unfished benthic	-	-
		Gymnothorax undulatus	unfished benthic	-	-
		Scuticaria tigrina	unfished benthic	-	-
Myliobatidae	*	*Aetobatus narinari*	noisy	-	-
Myliobatidae	*	*Manta birostris*	noisy	-	-
Oplegnathidae		*Oplegnathus punctatus*	fished	-	-
Ostraciidae		*Lactoria fornasini*	unfished benthic	-	-
		Ostracion meleagris	unfished benthic	-	-
		Ostracion whitleyi	unfished benthic	-	-
Pinguipedidae		*Parapercis schauinslandii*	unfished benthic	-	-
Pomacanthidae		*Apolemichthys arcuatus*	unfished benthic	-	-
Pomacanthidae		*Centropyge fisheri*	unfished benthic	-	-
Pomacanthidae		*Centropyge potteri*	unfished benthic	-	-
Pomacentridae		*Abudefduf abdominalis*	unfished midwater	-	-
		Abudefduf sordidus	unfished benthic	-	-
		Abudefduf vaigiensis	unfished midwater	-	-
		Chromis acares	noisy	-	-
		Chromis agilis	unfished midwater	-	-
		Chromis hanui	unfished benthic	-	-
		Chromis leucura	unfished benthic	-	-
		Chromis ovalis	unfished midwater	-	-
		Chromis vanderbilti	noisy	-	-
		Chromis verater	unfished midwater	-	-
		Dascyllus albisella	unfished midwater	-	-
		Plectroglyphidodon imparipennis	unfished benthic	-	-
		Plectroglyphidodon johnstonianus	unfished benthic	-	-
		Plectroglyphidodon sindonis	unfished benthic	-	-
		Stegastes fasciolatus	unfished benthic	-	-
Priacanthidae		*Priacanthus meeki*	fished	-	-
Ptereleotridae		*Ptereleotris heteroptera*	unfished benthic	-	-
Scaridae	*	*Calotomus carolinus*	fished	parrotfish	20
	*	*Calotomus zonarchus*	fished	parrotfish	20
	*	*Chlorurus perspicillatus*	fished	parrotfish	20
	*	*Chlorurus sordidus*	fished	parrotfish	20
		Scaridae spp.	fished	parrotfish	20
		Scarus dubius	fished	parrotfish	20
	*	*Scarus psittacus*	fished	parrotfish	20
	*	*Scarus rubroviolaceus*	fished	parrotfish	20
		Scarus sp	fished	parrotfish	20
Scorpaenidae		*Scorpaenopsis cacopsis*	unfished benthic	-	-
		Scorpaenopsis diabolus	unfished benthic	-	-

		Scorpaenopsis sp	unfished benthic	-	-
		Sebastapistes ballieui	unfished benthic	-	-
		Sebastapistes coniorta	unfished benthic	-	-
Serranidae	*	*Cephalopholis argus*	fished	grouper	20
		Pseudanthias bicolor	unfished benthic	-	-
		Pseudanthias thompsoni	unfished benthic	-	-
Sphyraenidae		*Sphyraena barracuda*	noisy	-	-
Synodontidae		*Synodontidae*	unfished benthic	-	-
Tetraodontidae		*Canthigaster amboinensis*	unfished benthic	-	-
		Canthigaster coronata	unfished benthic	-	-
		Canthigaster epilampra	unfished benthic	-	-
		Canthigaster jactator	unfished benthic	-	-
Zanclidae		*Zanclus cornutus*	unfished benthic	-	-
